# Comparison between Central and Brachial Blood Pressure in Hypertensive Elderly Women and Men

**DOI:** 10.1155/2017/6265823

**Published:** 2017-09-20

**Authors:** Bruno Bordin Pelazza, Sebastião Rodrigues Ferreira Filho

**Affiliations:** Postgraduate Program in Health Sciences, The Federal University of Uberlândia, Uberlândia, MG, Brazil

## Abstract

**Aim:**

To compare the values of central and brachial systemic blood pressure (SBP) between women and men over 60 years of age with systemic arterial hypertension.

**Methods:**

This study was a quantitative, descriptive, cross-sectional study with elderly patients admitted to and selected from spontaneous and scheduled demand at basic health units in Uberlândia, Minas Gerais, Brazil, between March 2013 and March 2014. We included 69 study participants and compared central and brachial SBP using a Sphygmocor® XCEL device (AtCor Medical, Sydney, Australia).

**Results:**

Significant differences were found in the blood pressure values of the whole population in the central versus brachial systolic blood pressure (SP) [140(21) versus 153(23) mmHg] and in the central versus brachial pulse pressure (PP) [55(18) versus 70(18) mmHg]. Additionally, females exhibited higher blood pressure levels than males [central SP 144(23) versus 134(16) mmHg and brachial SP 161(26) versus 148(18) mmHg and central PP 62(17) versus 45(14) mmHg and brachial PP 80(21) versus 63(15) mmHg, resp.].

**Conclusion:**

Elderly women exhibited higher blood pressure values than elderly hypertensive men.

## 1. Introduction

Systemic blood pressure (SBP) changes with ageing. Systolic blood pressure (SP) rises progressively, whereas diastolic blood pressure (DP) rises until the 6th decade of life and then stabilizes or gradually decreases [1–4].

SBP levels measured indirectly through cuffs placed either on the upper limbs or at the aortic root [the central SBP levels (SBPc)] constitute independent factors for cardiovascular risk (CVR) [5, 6]. Evidence has shown that SBPc, compared with brachial SBP (SBPb), is more closely associated with the injury of target organs [6–15]. Among the SBP components, Jankowski et al. [9] demonstrated that SP and pulse pressure (PP) were early and independent markers of CVR, whereas other studies described PP as a new marker of cardiovascular disease (CVD) [8–12]. In the elderly of both sexes, progressive arterial stiffness and early reflection waves amplify PP and elevate SP [6, 16]. Other contributors to vascular stiffness also exist, especially in women.

Epidemiological studies have confirmed that the physiological hormonal changes that occur in women during and after menopause affect the systemic circulation, thereby increasing SBP and consequently CVR. Additionally, CVD is the leading cause of mortality in postmenopausal women, with a prevalence equal to or higher than the prevalence in men due to low plasma oestrogen levels, among other factors [17–20].

As good markers of CVR, central and peripheral blood pressure values could diverge in the same patient and behave differently in elderly female patients. Therefore, the objective of the present study is to compare the central and brachial SBP values between women and men over 60 years of age with systemic arterial hypertension (SAH).

## 2. Materials and Methods

### 2.1. Population and Study Design

This study was a quantitative, descriptive, cross-sectional study with elderly patients ≥60 years of age who were admitted and selected from the spontaneous and scheduled demand at basic health units (BHUs) in the municipal network of Uberlândia, Minas Gerais, Brazil, from March 2013 to March 2014. The first 69 hypertensive participants were included in the study and were divided into three groups of patients as follows: (I) overall study population: 70 ± 7.3 years, *n* = 69; (II) women: 71 ± 7 years, *n* = 39; and (III) men: 68 ± 6.5 years, *n* = 30. All patients were active, stable, and conscious.

This study was conducted in accordance with the attributes defined in Resolution CNS 466/12 and approved by the Ethics Committee in Research of the Federal University of Uberlândia under opinion number 224,540 and CAAE 37440114.3.0000.5152.

### 2.2. SBP Measurement

Patients with SAH, a SBPb ≥ 140/90 mmHg, or a SBPb ≤ 140/90 mmHg with the use of one or more antihypertensive drugs (controlled) were considered.

A Sphygmocor XCEL applanation tonometry device (AtCor Medical, Sydney, Australia) was used to measure both SBPb and SBPc simultaneously and noninvasively. The components of the SBP surveyed were the SP, DP, PP, and mean arterial pressure (MAP) (both central and brachial). All values are expressed in mmHg. The SBPc and SBPb measurement procedure was performed only once and was based on a previous description [21].

### 2.3. Statistical Analysis

**Figure 1:**
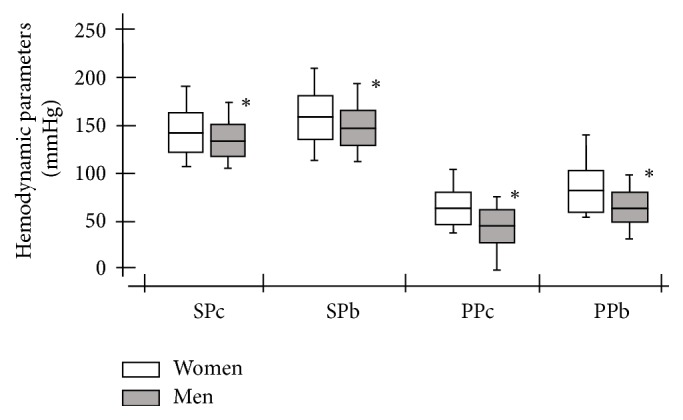
Comparison of systolic blood pressure and pulse pressure (both central and brachial) between the women and men. SPc = central systolic blood pressure; SPb = brachial systolic blood pressure; PPc = central pulse pressure; PPb = brachial pulse pressure. ^**∗**^Comparison of blood pressure parameters between all of the women and men (*P* < 0.05).

The results for the continuous variables shown in Tables 1 and 2 and Figure 1 are expressed as the mean ± standard deviation or as a proportion or percentage. First, we used BioEstat 3.0 to perform the D'Agostino-Pearson normality test. Subsequently, Student's *t*-test was applied to compare the values of the SBPc and SBPb components according to the normality of the sample. Differences were considered significant when *P* < 0.05. Statistical analysis was performed using Statistical Package for Social Science (SPSS) version 20 for Windows.

## 3. Results

The clinical characteristics of the overall study population and the population according to sex are shown in Table 1. Table 1 also showed that women used significantly more diuretics than did men.

Table 2 shows the hemodynamic variables of the overall population and the respective comparisons between the women and men.

Figure 1 shows the SP and PP variables (both central and brachial) between the women and men.

## 4. Discussion

Cross-sectional and longitudinal studies have confirmed a higher prevalence of hypertension in elderly postmenopausal women [17–19, 22] and greater arterial stiffening compared to that in men [20, 23]. Additionally, Piskorz and Brzostek [24] reported that postmenopausal women, compared with premenopausal women, had higher CVR and mortality [25]. One explanation for this finding is the possible decrease in the plasma oestrogen concentration, which exerts a protective effect against atherogenesis, atherosclerotic plaque formation, and, consequently, clinical manifestations of CVD [17–20].

In the present study, all patients were randomly included. We observed that women presented high SPc and PPc mean values, whereas men remained within the limits considered normal [5, 26]. We observed that the SPb and PPb values were increased in both sexes but were still higher in women (Table 2 and Figure 1).

Both SP and PP increase with age, mainly due to the decrease in elasticity of the large vessels as a consequence of their respective structural modifications [1, 3, 4, 20]. We found that neither the central nor brachial MAP values were significant in either sex (*P* < 0.05).

Son et al. [19] conducted a study with 95 female patients with a mean age of 52 years and found that indirectly measured SBPb was significantly higher in postmenopausal period than in premenopausal women; additionally, the number of SAH cases increased with advancing age. In our study, the components of both central and brachial SBP were compared. We found that the SP and PP levels in the women were higher than those in the men. The hypertensive postmenopausal women in the study by Son et al. were younger than the women in the present study; thus, our data indicate that the observed difference in blood pressure levels between men and women may persist at more advanced ages than observed by other authors.

Kim et al. [27] also showed that older women had higher PPb values compared to premenopausal levels, but the authors did not measure the central pressures or compare the values with men. Other studies [8, 12] have confirmed that women and men over 60 years of age with PPc ≥ 50 mmHg exhibit an increase in cardiovascular risk/events. Because the mean PPc value in women was 62 mmHg compared to 45 mmHg in males, women were assumed to have a higher CVR than men in similar age groups. We observed that the brachial SP and PP were higher than the central values in the overall study population. This finding may be attributed to arterial stiffening and the premature return of the reflected wave in systole (Table 2 and Figure 1) [9, 28, 29].

**Figure 2:**
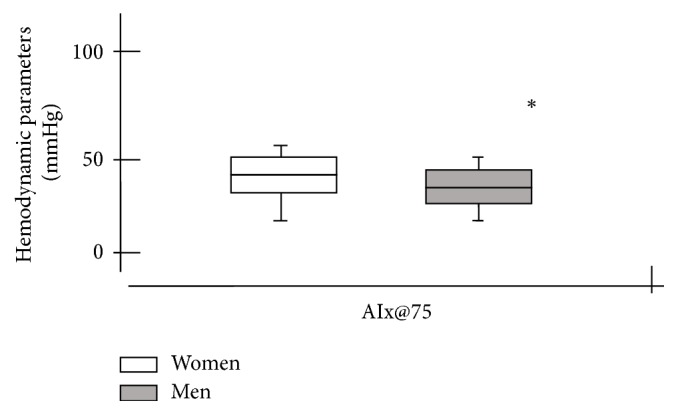
Comparison of heart rate-corrected augmentation indices between women and men. AIx@75 = heart rate-corrected augmentation index. ^**∗**^Comparison of blood pressure parameters between women and men (*P* < 0.05).

The AIx@75 (heart rate-corrected augmentation index) is considered as a composite marker of reflection waves and arterial stiffness. In this sense, it was evidenced that postmenopausal women had higher arterial stiffness when compared to elderly hypertensive men (Figure 2) [30].

To the best of our knowledge, Brazilian studies demonstrating and comparing the components of central and brachial SBP in postmenopausal women with elderly hypertensive men via applanation tonometry are rare. Notably, this study is a preliminary study with the inclusion of the first 69 hypertensive patients, which will help guide future research.

## 5. Limitations of the Study

This study is a cross-sectional study with the inherent limitations of this type of design. The study patients used several classes of antihypertensive drugs, including beta-adrenergic blockers (prescribed to 33% of the overall population), which do not effectively reduce central pressure levels [11–13, 31]. Additionally, the differences in the central and brachial SBP values found may be specific to the first 69 hypertensive patients analysed. Moreover, some patients examined may have exhibited acute SBP elevations due to stress during SBP measurement.

## 6. Conclusion

Significant differences were found in both the central and brachial SP and PP values between women and men. The elderly women presented higher blood pressure values than those of the elderly hypertensive men.

## Figures and Tables

**Table 1:** Clinical characteristics of the groups and the number of antihypertensive agents.

Parameters	Overall population (*n* = 69)	Women (*n* = 39)	Men (*n* = 30)
Age	70 (7)	71 (7)	68 (7)
Sedentary lifestyle (%)	62	62	63
Smoking (%)	14	10	20
Diabetes (%)	35	33	37
Weight (kg)	79 (17)	76 (17)	83 (16)
Height (cm)	1.6 (0.1)	1.5 (0.1)^*∗*^	1.7 (0.1)^*∗*^
WC (cm)	102 (16)	102 (15)	103 (17)
BMI	31 (6)	32 (8)	30 (5)
Antihypertensive drugs (*n*)	1.6 (1.3)	1.8 (1.3)	1.4 (1.3)
BB (%)	33	38	27
CCB (%)	13	10	17
ARB (%)	35	36	33
Vasodilator (%)	3	3	3
ACEI (%)	30	31	30
Diuretics (%)	41	49^*∗*^	30^*∗*^
Controlled hypertensives (%)	41	41	40

*∗* = comparison between women and men (*P* < 0.05); ( ) = standard deviation; WC = waist circumference; BMI = body mass index; *n* = number; % = percentage; kg = kilogram; cm = centimetres; BB = beta-adrenergic blocker; CCB = calcium channel blocker; ARB = angiotensin receptor blocker; ACEI = angiotensin-converting enzyme inhibitor.

**Table 2:** Characteristics of the hemodynamic parameters of the groups.

Parameters	Overall population (*n* = 69)	Women (*n* = 39)	Men (*n* = 30)
HR (bpm)	74 (19)	74 (20)	73 (19)
RR (bpm)	17 (3)	17 (4)	17 (3)
SPc versus SPb (mmHg)	140 (21)/153 (23)^†^	—	—
DPc versus DPb (mmHg)	84 (15)/83 (15)	—	—
PPc versus PPb (mmHg)	55 (18)/70 (18)^†^	—	—
MAPc versus MAPb (mmHg)	103 (15)/106 (16)	—	—
SPc (mmHg)	—	144 (23)^*∗*^	134 (16)^*∗*^
DPc (mmHg)	—	82 (15)	88 (15)
PPc (mmHg)	—	62 (17)^*∗*^	45 (14)^*∗*^
MAPc (mmHg)	—	103 (16)	103 (14)
SPb (mmHg)	—	161 (26)^*∗*^	148 (18)^*∗*^
DPb (mmHg)	—	81 (15)	86 (15)
PPb (mmHg)	—	80 (21)^*∗*^	63 (15)^*∗*^
MAPb (mmHg)	—	106 (17)	107 (14)
AIx@75 (%)	—	41 (12)^*∗*^	30 (8)^*∗*^

^†^Comparison between the parameters of the entire population (*P* < 0.05); ^*∗*^comparison between parameters of the women and men (*P* < 0.05); % = percentage; ( ) = standard deviation; HR = heart rate; RR = respiratory rate; c = central; b = brachial; bpm = beats per minute; bpm = breaths per minute; mmHg = millimetres of mercury; SP = systolic blood pressure; DP = diastolic blood pressure; PP = pulse pressure; MAP = mean arterial pressure; and AIx@75 = heart rate-corrected augmentation index.
